# Supplementary material for: Predicting risk of late age-related macular degeneration using deep learning
Source: NPJ Digit Med. 2020 Aug 27;3:111. doi: 10.1038/s41746-020-00317-z (PMC7453007; doi:10.1038/s41746-020-00317-z)
Supplement: Supplementary file 1 — Supplementary Information [file 41746_2020_317_MOESM1_ESM.pdf]

a

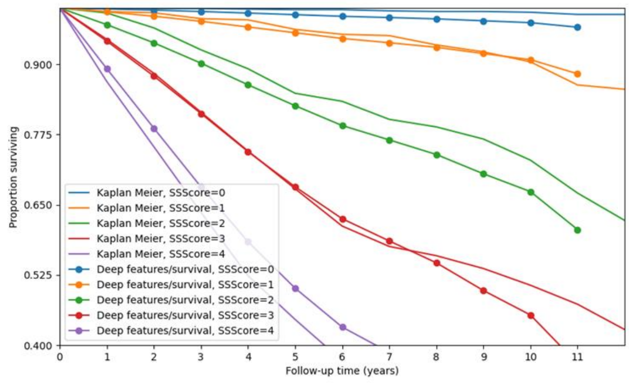

b

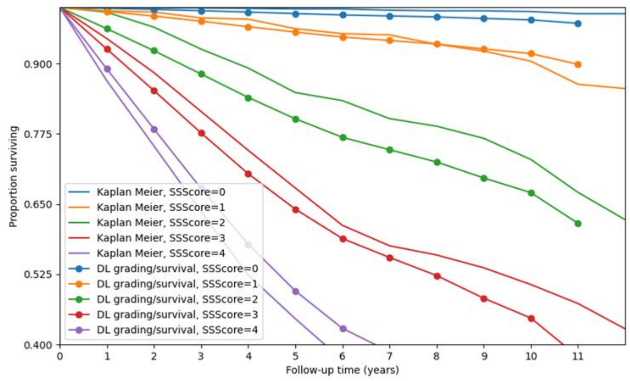

c

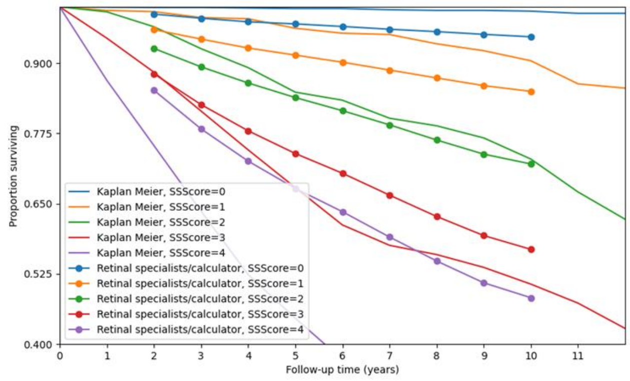

Supplementary Figure 1. Survival model predictions for the 5 groups. The participants were split into 5 groups based on the AREDS simplified severity scores at the baseline. Actual survival for the 5 groups is shown in lines (Kaplan-Meier curves). The deep features/survival, DL grading/survival, and retinal specialist/survival model predictions for the five groups are shown in lines with markers. The deep features/survival predictions corresponded better to actual progression data than those of the other two models.

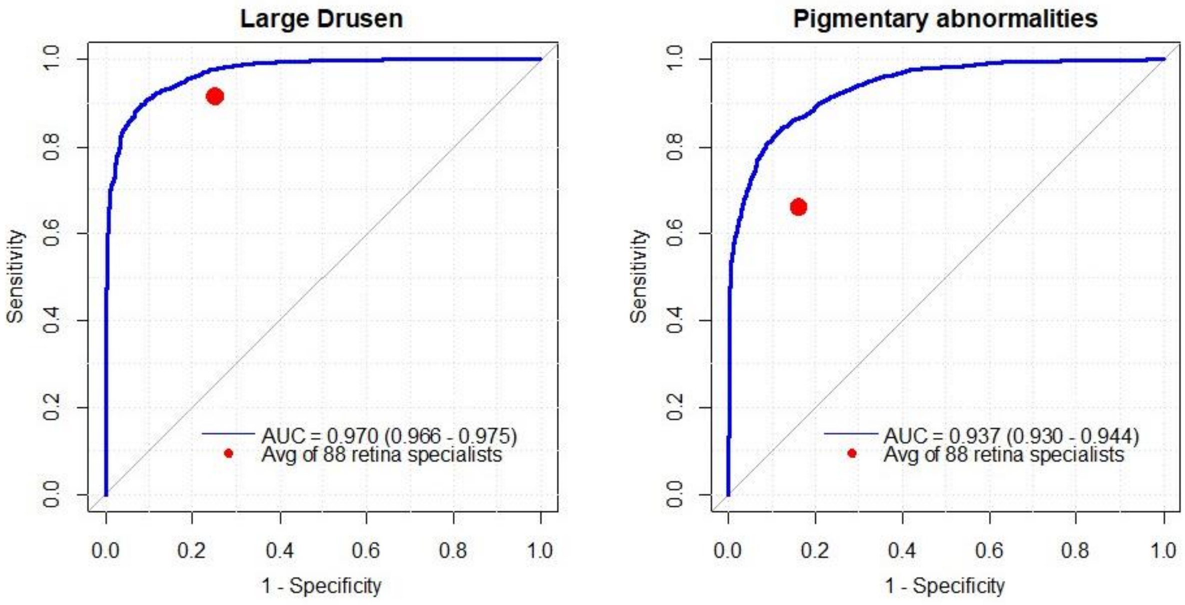

Supplementary Figure 2. Receiver operating characteristic curves for the deep neural networks to grade drusen size and pigmentary abnormalities presence/absence on the combined AREDS/AREDS2 test sets (601 participants). The AUC was used to evaluate the performance of deep neural networks in image classification (as opposed to predictions of progression), and for comparison with the retinal specialists (with reference to the Reading Center grades as the ground truth).

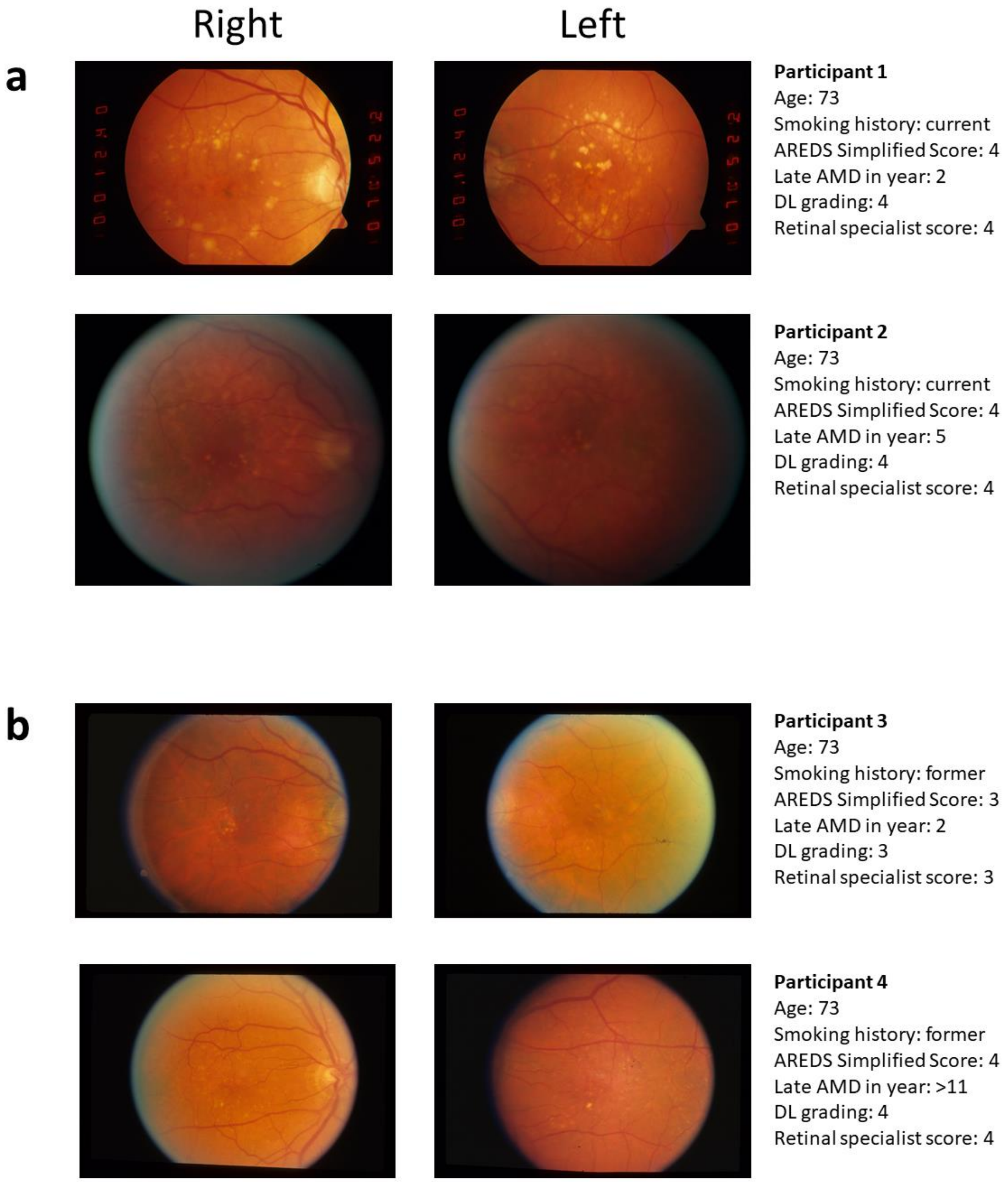

Supplementary Figure 3. Example cases of progression to late age-related macular degeneration (AMD) where deep features/survival made more accurate predictions than retinal specialist/calculator or retinal specialist/Simplified Severity Scale (SSS). a. Both participants were the same age (73 years) and had the same smoking history (current) and AREDS SSS scores (4) at baseline. The retinal specialist graded the SSS correctly. However, participant 1 progressed to late AMD at year 2 (GA in the right eye), but participant 2 progressed to late AMD at year 5 (GA in the left eye). Hence, both the retinal specialist/calculator and retinal specialist/SSS approaches *incorrectly* assigned the same risk

of progression to both participants (0.413). However, the deep feature/survival approach *correctly* assigned a higher risk of progression to participant 1 (0.751) and a lower risk to participant 2 (0.591). b. Both participants were the same age (73 years) and had the same smoking history (former). Their AREDS SSS scores at baseline were 3 and 4, respectively. The retinal specialist graded the SSS correctly. However, participant 3 progressed to late AMD at year 2 (neovascular AMD in the right eye), while participant 4 had still not progressed to late AMD by final follow-up at year 11. Hence, both the retinal specialist/calculator and retinal specialist/SSS approaches *incorrectly* assigned a lower risk of progression to participant 3 (0.259) and a higher risk to participant 4 (0.413). However, the deep feature/survival approach *correctly* assigned a higher risk of progression to participant 4 and a lower risk to participant 3 (0.569 vs 0.528).

Supplementary Table 1. Number of images used to develop the classification network.

Image characteristics	AREDS	AREDS2
Number of images	57,375	17,658
Training	40,455	12,391
Development	5,535	1,738
Test	11,385	3,529
AMD drusen feature, by image, as classified by Reading Center		
No drusen or small drusen, No. (%)	24,290	191
Medium drusen, No. (%)	15,464	921
Large drusen, No. (%)	17,621	16,546
AMD pigmentary abnormalities, by image, as classified by Reading Center		
absent, No. (%)	40,738	3,855
present, No. (%)	16,637	13,803

Supplementary Table 2. Multivariate Association of Phenotypic, Demographic, and Genetic Variables and Progression to late AMD. Deep features with p value<0.05 are shown.

DL grading/survival				Deep Feature/survival			
Variable	Hazard ratio	95% CI	p-value	Variable	Hazard ratio	95% CI	p-value
Age	1.05	1.04-1.07	<.001	Age	1.05	1.04-1.06	<.001
Smoking status	1.17	1.03-1.32	0.017	Smoking status	1.14	1.00-1.30	0.055
CFH rs1061170	0.95	0.83-1.07	0.382	*CFH* rs1061170	0.89	0.77-1.04	0.085
ARMS2 rs10490924	0.95	0.83-1.09	0.490	*ARMS2* rs10490924	0.90	0.77-1.04	0.161
AMD GRS	1.35	1.24-1.47	<.001	AMD GRS	1.38	1.25-1.52	<.001
drusen score (LE)	2.26	1.84-2.78	<.001	feature335	0.14	0.05-0.37	<.001
drusen score (RE)	1.89	1.56-2.28	<.001	feature79	0.24	0.09-0.66	0.005
pig abn (LE)	2.28	1.88-2.76	<.001	feature449	0.56	0.35-0.91	0.020
pig abn (RE)	1.72	1.42-2.08	<.001	feature234	9.41	1.04-85.11	0.046

LE-left eye, RE-right eye, pig abn-pigmentary abnormality, GRS-Genetic Risk Score.

Supplementary Table 3. The C-statistic (95% confidence interval) of the survival models in predicting risk of progression to late age-related macular degeneration on the combined AREDS/AREDS2 test sets (601 participants).

**Models**	**1**	**2**	**3**	**4**	**5**
Late AMD					
DL grading /calculator	-	82.2(81.8,82.6)	82.6(82.3,83.0)	83.4(83.1,83.6)	83.5(83.3,83.7)
DL grading /SSS*	-	-	-	-	82.3(82.1,82.5)
Geographic atrophy					
DL grading /calculator	**-**	84.7(84.4,85.1)	83.4(83.1,83.7)	84.2(83.9,84.4)	83.7(83.4,83.9)
DL grading /SSS*	-	-	-	-	-
Neovascular AMD					
DL grading /calculator	-	78.2(77.4,79.0)	80.1(79.5,80.6)	78.7(78.3,79.1)	79.4(79.1,79.7)
DL grading /SSS*	-	-	-	-	-

Supplementary Table 4. The C-statistic (95% confidence interval) of three models in predicting risk of progression to late age-related macular degeneration on the combined AREDS/AREDS2 test sets (601 participants).

	**DL grading**	**DL features**
Nnet-survival	0.803 (0.799, 0.808)	0.851 (0.846, 0.856)
DeepSurv	0.842 (0.837, 0.847)	0.859 (0.854, 0.865)
COX PH	0.849 (0.846, 0.853)	0.867 (0.865, 0.868)
