# Supplementary material for: Predicting risk of late age-related macular degeneration using deep learning
Source: NPJ Digit Med. 2020 Aug 27;3:111. doi: 10.1038/s41746-020-00317-z (PMC7453007; doi:10.1038/s41746-020-00317-z)
Supplement: Supplementary file 2 — Reporting Summary [file 41746_2020_317_MOESM2_ESM.pdf]

nature research

Corresponding author(s): Zhiyong Lu, Emily Chew

Last updated by author(s): Apr 30, 2020

Reporting Summary

Nature Research wishes to improve the reproducibility of the work that we publish. This form provides structure for consistency and transparency in reporting. For further information on Nature Research policies, see Authors & Referees and the Editorial Policy Checklist.

Statistics

For all statistical analyses, confirm that the following items are present in the figure legend, table legend, main text, or Methods section.

n/a	Confirmed	
☐	☒	The exact sample size (*n*) for each experimental group/condition, given as a discrete number and unit of measurement
☐	☒	A statement on whether measurements were taken from distinct samples or whether the same sample was measured repeatedly
☐	☒	The statistical test(s) used AND whether they are one- or two-sided<br>*Only common tests should be described solely by name; describe more complex techniques in the Methods section.*
☐	☒	A description of all covariates tested
☒	☐	A description of any assumptions or corrections, such as tests of normality and adjustment for multiple comparisons
☐	☒	A full description of the statistical parameters including central tendency (e.g. means) or other basic estimates (e.g. regression coefficient) AND variation (e.g. standard deviation) or associated estimates of uncertainty (e.g. confidence intervals)
☐	☒	For null hypothesis testing, the test statistic (e.g. *F*, *t*, *r*) with confidence intervals, effect sizes, degrees of freedom and *P* value noted<br>*Give P values as exact values whenever suitable.*
☒	☐	For Bayesian analysis, information on the choice of priors and Markov chain Monte Carlo settings
☒	☐	For hierarchical and complex designs, identification of the appropriate level for tests and full reporting of outcomes
☒	☐	Estimates of effect sizes (e.g. Cohen's *d*, Pearson's *r*), indicating how they were calculated

Our web collection on statistics for biologists contains articles on many of the points above.

Software and code

Policy information about availability of computer code

Data collection	Python
Data analysis	The model training was done using Tensorflow, Keras, and Python. Code availability stated in the manuscript.

For manuscripts utilizing custom algorithms or software that are central to the research but not yet described in published literature, software must be made available to editors/reviewers. We strongly encourage code deposition in a community repository (e.g. GitHub). See the Nature Research guidelines for submitting code & software for further information.

Data

Policy information about availability of data

All manuscripts must include a data availability statement. This statement should provide the following information, where applicable:

- Accession codes, unique identifiers, or web links for publicly available datasets
- A list of figures that have associated raw data
- A description of any restrictions on data availability

The AREDS dataset generated during and/or analyzed during the current study is available in the dbGAP repository, https://www.ncbi.nlm.nih.gov/projects/gap/cgi-bin/study.cgi?study_id=phs000001.v3.p1. The AREDS2 dataset generated during and/or analyzed during the current study is not publicly available due to it being under IRB review but is available from the corresponding author on reasonable request.

Field-specific reporting

Please select the one below that is the best fit for your research. If you are not sure, read the appropriate sections before making your selection.

☒ Life sciences ☐ Behavioural & social sciences ☐ Ecological, evolutionary & environmental sciences

For a reference copy of the document with all sections, see nature.com/documents/nr-reporting-summary-flat.pdf

Life sciences study design

All studies must disclose on these points even when the disclosure is negative.

Sample size	We collected 2177 participants from AREDS and 1121 participants from AREDS2.
Data exclusions	Participants were excluded if they had late AMD in either eye at baseline or had missing data for any of the study variables.
Replication	We have retrained and tested our models several times and got consistent results. Our model was also tested on an external dataset the model has not seen during training. We have also calculated 95% Confidence Intervals for our test results.
Randomization	We used random sampling to split our data into train, validation and test set, but care was taken to ensure that there was no patient overlap between training, validation and test sets.
Blinding	Investigators are blinded to group allocation during data collection and analysis.

Reporting for specific materials, systems and methods

We require information from authors about some types of materials, experimental systems and methods used in many studies. Here, indicate whether each material, system or method listed is relevant to your study. If you are not sure if a list item applies to your research, read the appropriate section before selecting a response.

Materials & experimental systems

n/a	Involved in the study	
☒	☐	Antibodies
☒	☐	Eukaryotic cell lines
☒	☐	Palaeontology
☒	☐	Animals and other organisms
☐	☒	Human research participants
☐	☒	Clinical data

Methods

n/a	Involved in the study	
☒	☐	ChIP-seq
☒	☐	Flow cytometry
☒	☐	MRI-based neuroimaging

Human research participants

Policy information about studies involving human research participants

Population characteristics	The population characteristics are shown in Table 1.
Recruitment	Participants were recruited from outpatient clinics of retinal specialists in both academic and community settings
Ethics oversight	Institutional Review Boards (IRBs) of each of the clinical sites approved of the study protocol and each participant provided written informed consent

Note that full information on the approval of the study protocol must also be provided in the manuscript.

Clinical data

Policy information about clinical studies

All manuscripts should comply with the ICMJE guidelines for publication of clinical research and a completed CONSORT checklist must be included with all submissions.

Clinical trial registration	NCT00000145, NCT00345176
Study protocol	https://www.nei.nih.gov/research/clinical-trials/age-related-eye-disease-studies-aredsareds2
Data collection	Details of the AREDS dataset can be found at: https://www.ncbi.nlm.nih.gov/projects/gap/cgi-bin/study.cgi?study_id=phs000001.v3.p1

Outcomes	Development and evaluation of techniques for computer aided AMD prognosis from color fundus photograph would potentially benefit patients.
